# Emphysematous cystitis treated with targeted antibiotic therapies: a report of two cases

**DOI:** 10.3389/fmed.2026.1821972

**Published:** 2026-08-31

**Authors:** Quanbo Yu, Yanhui Wang, Xinqi Zhang

**Affiliations:** 1 Department of Hand Surgery, The 960th Hospital of the PLA Joint Logistics Support Force, Jinan, Shandong, China; 2 Department of Emergency, The 960th Hospital of the PLA Joint Logistics Support Force, Jinan, Shandong, China

**Keywords:** bladder pneumatosis, case report, diabetes mellitus, emphysematous cystitis, urinary tract infection

## Abstract

Emphysematous cystitis (EC) is a rare, complicated urinary tract infection characterized by gas formation in and around the bladder wall, often occurring in older adults with diabetes mellitus, urinary tract obstruction, or long-term indwelling urinary catheters. EC can be easily misdiagnosed due to atypical symptoms and signs. Delayed treatment causes life-threatening complications like emphysematous pyelonephritis, bladder perforation, or septic shock. Here, we retrospectively analyzed two cases of EC who had been effectively treated and discharged following antibiotics, strict blood sugar control, bladder irrigation, and symptomatic and supportive treatment. Through literature review, we summarized the clinical characteristics, diagnostic skills, and therapeutic strategies for EC. Our experience may inform the early diagnosis and standardized management to improve the prognosis of EC.

## Introduction

Emphysematous urinary tract infection (EUTI) is a rare but serious urinary tract infection associated with gas formation in the kidneys, ureters, or bladder (1, 2). It can be classified into emphysematous pyelonephritis (EPN) and emphysematous cystitis (EC) according to the infection site. EC, as a rare and complicated condition of lower urinary tract infection, is occasionally described in case reports (3, 4). Lacking knowledge of this rare disease easily causes misdiagnosis and delayed treatment. Here, we summarized clinical characteristics, diagnostic skills, and therapeutic strategies for two cases of EC, and reviewed relevant literatures.

## Case presentation

### Case 1

A 73-year-old female was admitted on April 7, 2025, complaining of abdominal pain for 3 days. She was complicated with diabetes mellitus and cerebral infarction. She denied a history of abdominal trauma and urethral procedures and interventions. Physical examination showed a high blood pressure (175/68 mmHg) and normal temperature (36.5 °C), pulse (78 beats per minute) and respiratory rate (16 breaths per minute). The patient was in poor consciousness and mental state, lacking the ability to cooperate with the physical examination. Coarse crackles were auscultated in both lungs, without wet or dry rales. Cardiac auscultation results were normal, with a regular heart rhythm and no murmurs indicating pathological conditions of heart valves. Abdominal palpation showed a soft abdomen, no rebound tenderness, and no enlargement of the liver and spleen. However, a mild tenderness was elicited from the middle abdomen. No abnormalities were found in the nervous system, and no swelling was seen in lower extremities.

**FIGURE 1:**
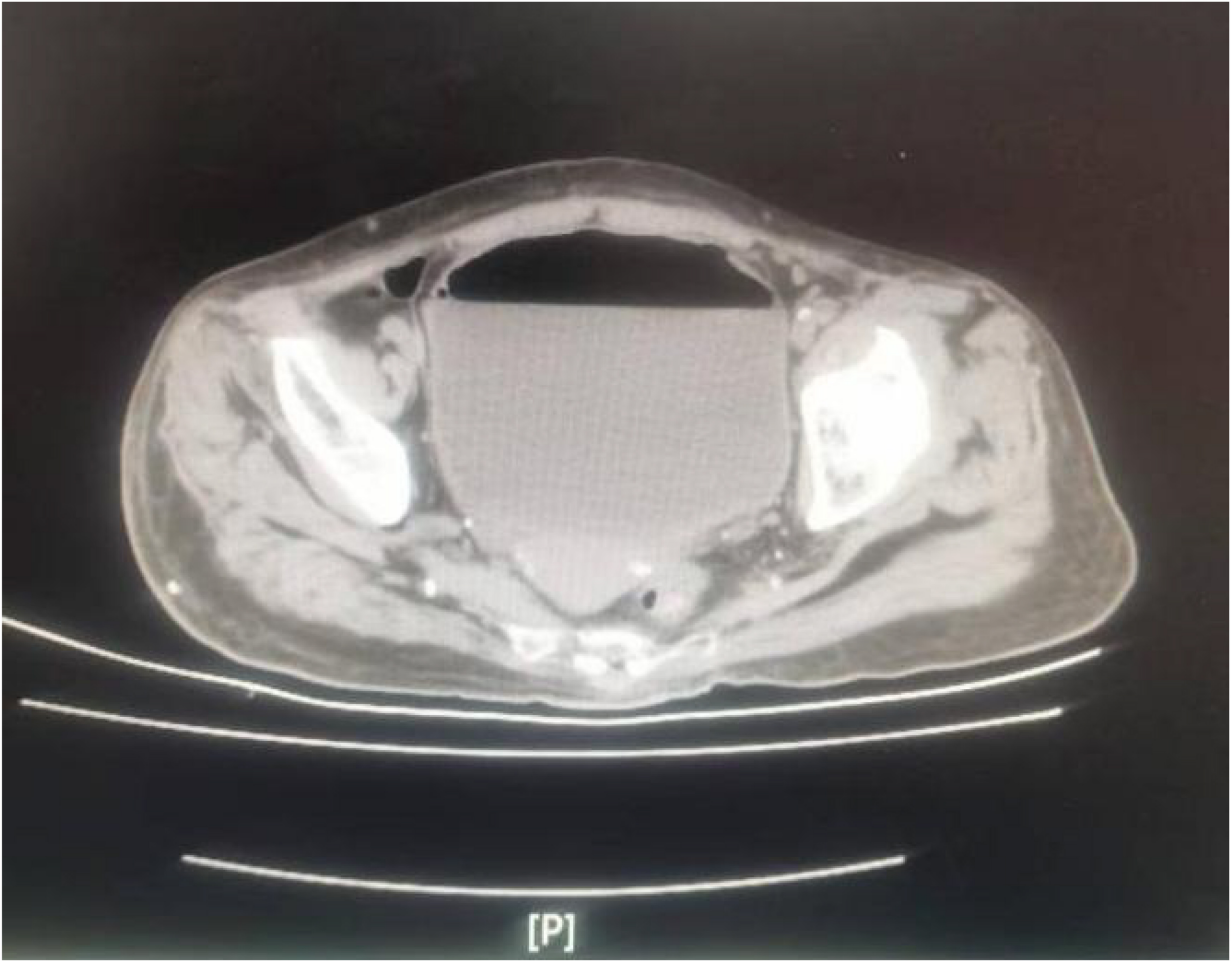
An air-liquid level in the bladder visualized on the abdominal CT scan on April 6, 2025.

The complete blood count (CBC) showed a reduced hemoglobin (Hb, 83 g/L) and normal reference ranges of white blood cell count (WBC, 5.19 × 10^9^/L) and platelet count (PLT, 251 × 10^9^/L). Liver function and biochemistry tests uncovered reduced albumin (ALB, 27.6 g/L) and serum potassium (K^+^, 2.59 mmol/L), as well as elevated blood sugar (12.5 mmol/L), procalcitonin (PCT, 0.07 ng/mL; normal reference range, <0.05 ng/mL), C-reactive protein (CRP, 16.68 mg/L), N-terminal pro-B-type natriuretic peptide (NT-proBNP, 2 531 ng/L), and glycosylated hemoglobin (HbA1C, 7.6%). Serum creatinine (Cr, 73 μmol/L) and sodium (Na^+^, 142.0 mmol/L) were normal. Thyroid function and cardiac enzyme test showed no obvious abnormalities. The urinalysis showed an extremely elevated count of leukocytes to 1 778.7 WBCs per high-powered field (HPF), and bacteria in the urine were quantified at 161.04 colony forming unites (CFU)/μL. Extended-spectrum beta-lactamase (ESBL)-producing *Escherichia coli*, which was sensitive to carbapenems, was detected in the urine culture. An air-liquid level in the bladder was visualized on the abdominal computed tomography (CT) scan (Figure 1).

**FIGURE 2:**
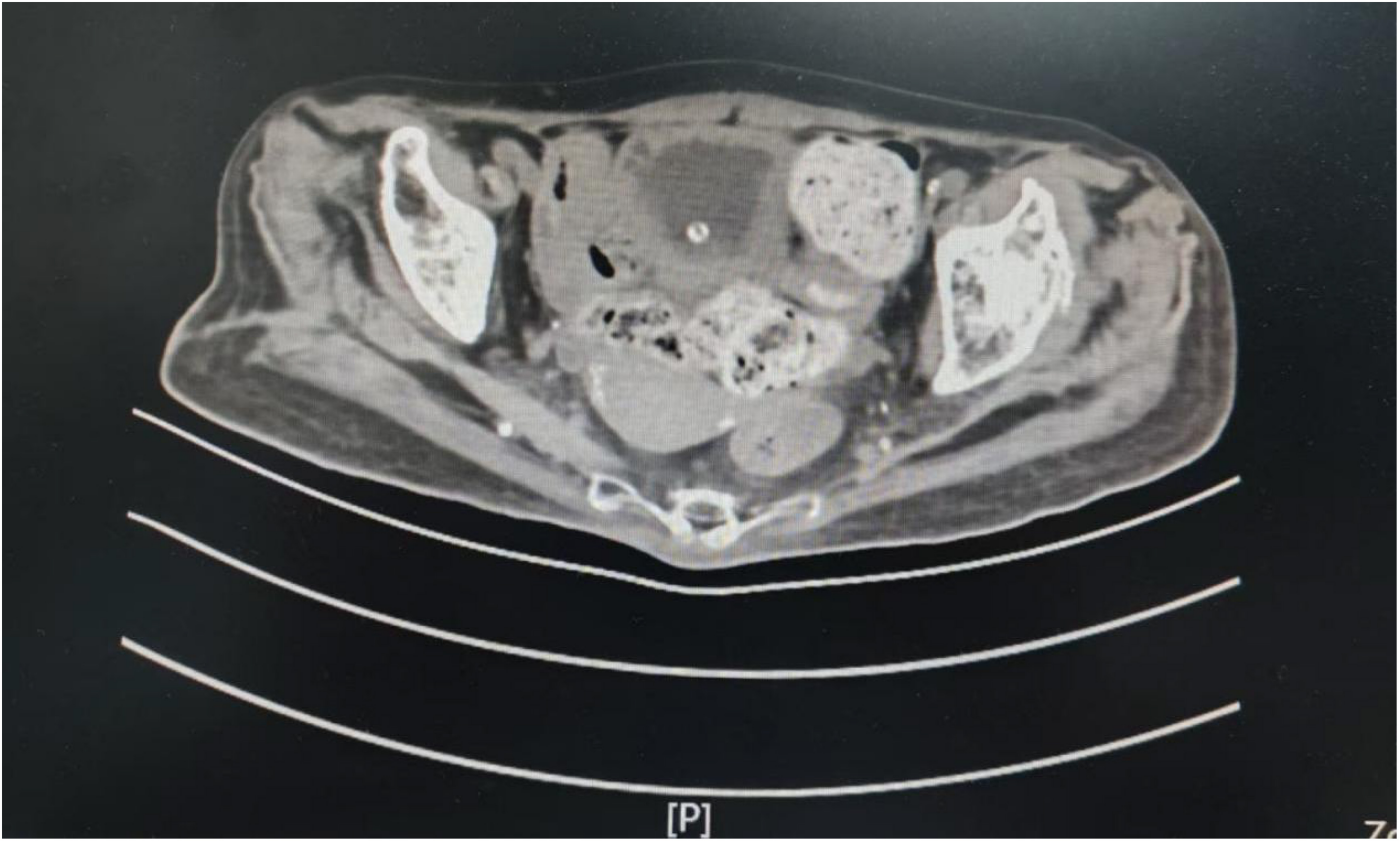
Thickened bladder wall without gas shadows on the re-examined abdominal CT scans on August 17, 2025.

Following comprehensive laboratory and imaging examinations, the patient was treated with intravenous administrations of 1.0 g meropenem every 8 h for 8 days, followed by a de-escalation antibiotic therapy via intravenous administrations of 3.0 g cefoperazone sulbactam every 8 h for 7 days. She was recovered and discharged, with post-discharge bladder irrigation and blood sugar control via an intensive insulin therapy. A follow-up urine culture at 3 weeks of discharge was negative, and the thickened bladder wall without gas shadows was seen on CT scans at 4 months (Figure 2).

### Case 2

An 80-year-old female presented on June 25, 2025 with 6 days of fever. She suffered from diabetes mellitus, hypertension, coronary heart disease, and chronic obstructive pulmonary disease (COPD). She was diagnosed with EC according to the active response to antibiotic treatment against abundant white and red blood cells in the urine culture and gas-producing pathogens detected in a mid-stream urine culture, as well as imaging evidence of massive gas shadows after 1 week of catheterization. We made a clear differentiation from iatrogenic gas introduction caused by a leaking urinary catheter for two possible reasons. First, iatrogenic gas introduction would not cause abnormal findings in urine culture and CBC. Second, gas shadows caused by air leaks of catheterization usually disappear within 3 days, leaving only a small amount of free air visible in the area where the urinary catheter advances.

Physical examination showed a high blood pressure (159/54 mmHg) and normal ranges of temperature (37.1 °C), pulse (86 beats per minute) and respiratory rate (17 breaths per minute). The patient had a clear mind, and was ready to cooperate. Breath sounds in both lungs were coarse, without an auscultation of wet or dry rales. Regular heartbeats were heard, with no pathological murmurs in each heart valve. A soft abdomen was palpated, without tenderness, rebound tenderness, or liver and spleen enlargement. Abnormalities in the nervous system and lower limb swelling were not detected.

**FIGURE 3:**
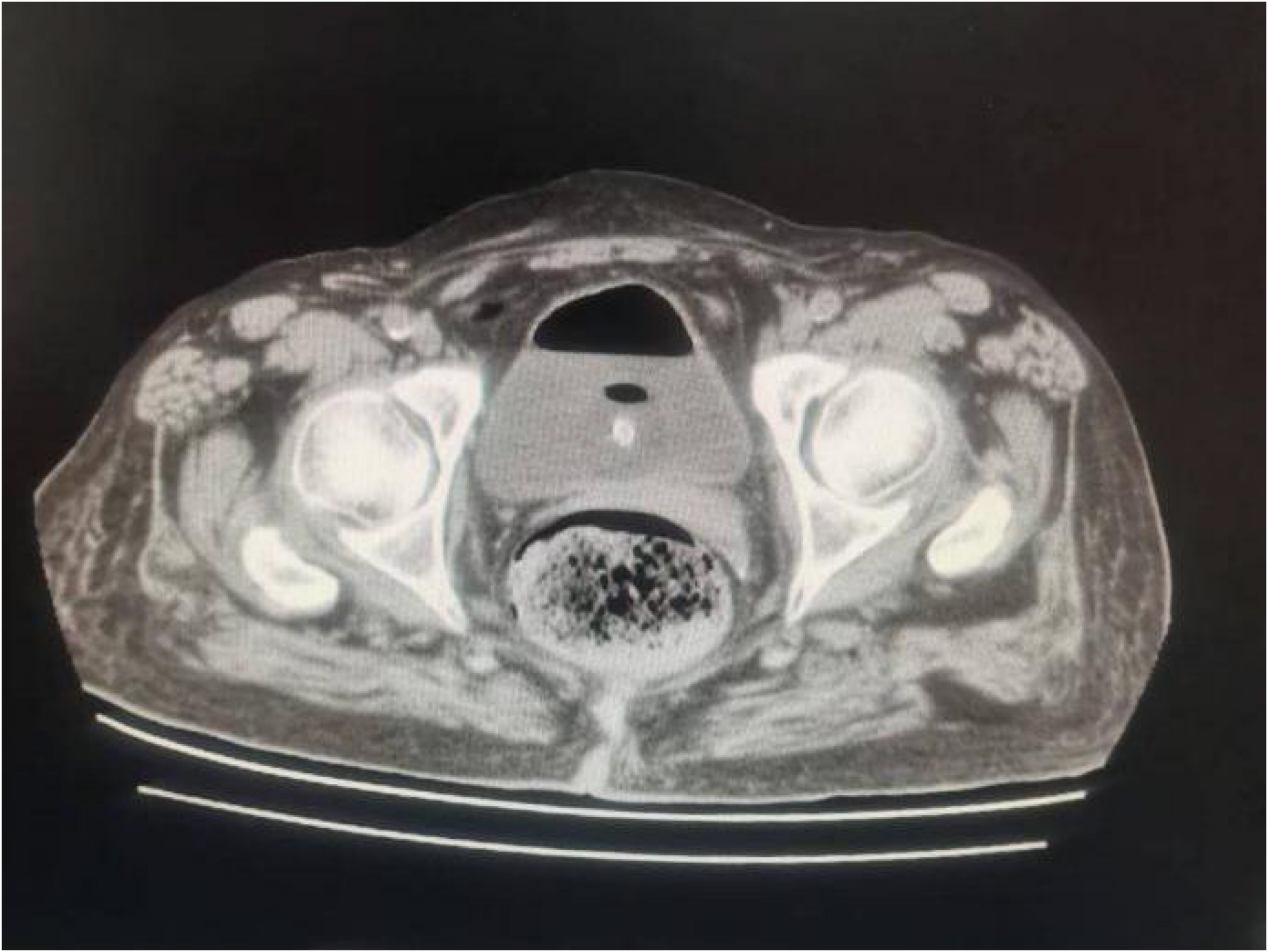
Gas shadows within the bladder visualized on the abdominal CT scan on July 3, 2025.

White blood cell (7.58 × 10^9^/L), Hb (117 g/L) and PLT (165 × 10^9^/L) were detected within normal references by the CBC. Elevated PCT (0.07 ng/mL; normal reference range, <0.05 ng/mL), NT-proBNP (872.0 ng/L), HbA1C (6.4%), and blood sugar (6.3 mmol/L), as well as a reduced Na^+^ (128.0 mmol/L) were detected. Other key biomechanical indicators were normal, including Cr (45 μmol/L), ALB (38.3 g/L), K^+^ (4.25 mmol/L), and CRP (<6.00 mg/L). Thyroid function, liver function, and cardiac enzyme testing did not show abnormalities. The urinalysis quantified 40.92 leukocytes per HPF, and 293.48 CFU/μL. *Klebsiella pneumoniae* carbapenemase (KPC)-producing strains that were sensitive to ceftazidime-avibactam were positive in the urine. Gas shadows within the bladder were visualized on the abdominal CT scan (Figure 3). Treatment against infections, blood sugar control by an intensive insulin therapy, and bladder irrigation were then given to her. Symptoms and signs were effectively alleviated following intravenous administrations of 2.5 g ceftazidime-avibactam every 8 h for 7 days, followed by 5-day intravenous administrations of 3.0 g cefoperazone sulbactam every 8 h. Her conditions were effectively improved and discharged. A negative urine culture was detected at 4 weeks of discharge, and since after, she has been lost to follow-up.

## Discussion

Emphysematous cystitis is a rare infectious disease initially proposed by Professor Bailey in 1961 (5). It frequently affects older women with diabetes mellitus. Statistics show that about 60% of EC patients are complicated with diabetes mellitus, and the incidence of EC is twice as high in women as in men. High-risk factors for EC consist of chronic urinary tract infection, long-term indwelling urinary catheters, neurogenic cystitis, and use of immunosuppressive drugs (6–8). Untreated timely, EC may aggravate into fatal complications like bladder rupture, bladder necrosis, EPN, and sepsis. Through literature review, the mortality of EC ranges from 7% to 14% (9). Generally, diabetes mellitus and gas-producing bacterial infections contribute to the onset of EC. *Escherichia coli* and *Klebsiella pneumoniae* are two Gram-negative bacteria that produce gas bubbles by fermenting glucose in the urine or tissue via glycolysis, as visualized by gas accumulation in the bladder on images. In individuals with diabetes mellitus, a high-sugar microenvironment inhibits the phagocytic function of neutrophils and offers glycolysis substrates for gas-producing bacteria, resulting in the formation of air pockets in and around the bladder wall (6, 7).

In this report, both EC patients were complicated with diabetes mellitus for a long period. Urine cultures identified ESBL-producing *Escherichia coli* in Case 1 and KPC-producing bacteria in Case 2, confirming the involvement of bacterial mechanisms in EC. Evidence has mounted to show the importance of bladder wall remodeling by chronic inflammation in the pathogenesis of EC, similarly to emphysema-induced destructive damages to the lungs. In Case 2, chronic urinary tract infection and bladder infection secondary to the long-term indwelling urinary catheter cause damages to the bladder mucosal barrier and rupture of elastic muscle fibers, leading to the formation of air-containing spaces. TRPV4 (transient receptor potential cation channel subfamily V member 4), a type of calcium-permeable channel that is widely expressed on the epithelium and smooth muscle of the bladder, function as crucial pathological sensors in the urinary bladder by detecting inflammatory signals. Stimulated by chronic inflammatory responses, TRPV4 is activated to induce mucosal edema and fibrous hyperplasia in the bladder wall, eventually decreasing the elasticity and urine storage function of the bladder (10, 11). This underscores the long-term management of chronic bladder inflammation in EC patients, even after the effective control of infections.

Clinical manifestations of EC are often non-specific, and predominated by abdominal pain and fever. A portion of EC patients complain of urinary tract symptoms like frequent and urgent urination, mimicking acute gastroenteritis and common cystitis (9, 12). This results in a high misdiagnosis rate of EC. Here, Case 2 was initially suspected of acute gastroenteritis for the major symptoms of nausea, vomiting, and difficulty urinating in the early stage, without typical manifestations of bladder irritations. She was finally diagnosed with EC by combined evidence of abdominal CT gas signs plus markedly abnormal urine routine microscopy and pathogen-positive urine culture, and a timely intervention effectively protected against severe complications. Abdominal CT, which can visualize gas in and around the bladder wall, is considered as the gold standard to make a diagnosis of EC. The “gas bubble sign” (bead-like air bubbles within the bladder cavity) and an air-liquid level in the bladder cavity are typical features on CT to effectively distinguish EC from other diseases of the urinary system. Ultrasound, however, is less effective in diagnosing EC due to its low sensitivity and the failure to detect gas signs. We therefore recommended CT as a priority to make a definite diagnosis of EC (13).

Conservative treatments, including anti-infection, bladder irrigation and management of underlying diseases, are preferred to EC. Bladder irrigation in EC patients is a procedure to reduce the pressure and gas accumulation within the bladder, prevent urine reflux, and discharge urine and purulent secretions (14). Both cases in the present study were indwelled three-way Foley catheters for irrigating the bladder using 500 mL of 0.9% sodium chloride at a rate of 80–100 mL/h. Continuous bladder irrigation (CBI) was conducted for 5–7 days until the returning fluid was clear and purulent secretions have completely resolved. Differing from CBI, clean intermittent catheterization (CIC) is a temporary procedure where a catheter is inserted multiple times a day to intermittently drain the bladder, rather a continuous irrigation. CBI, through continuous irrigation, was more suitable for the two cases of EC to remove accumulated gas, high-sugar urine and inflammatory exudates in the bladder, thus inhibiting the proliferation of gas-producing pathogens and avoiding further aggravation of bladder mucosal damage. In this study, local infusion of antibiotics was not used in the CBI. Systemic targeted antibiotics based on urine culture findings were effective to eliminate pathogenic bacteria. Existing evidence on clinical management of EC supports the use of 0.9% sodium chloride as the first-line local treatment. Bladder irrigation using antibiotics is only used for EC patients who are poorly responsive to systemic antibiotic treatment and with protracted and refractory bladder mucosal infection. A series of procedures was conducted in our study to minimize the risk of secondary infection caused by bladder irrigation, including strict procedures of nursing care for bladder catheterization, a regular replacement of drainage bags, and a closed urinary drainage system during bladder irrigation. Antibiotic treatment of EC should be given based on the findings of urine cultures and drug susceptibility tests, often commencing with broad-spectrum antibiotics and then tailored to targeted antibiotics (e.g., carbapenems for multi-resistant bacteria). Meanwhile, blood sugar in EC patients should be strictly controlled, thereby preventing disease aggravation within a high-sugar microenvironment and creating a stable internal environment for underlying disease management. Both the two reported cases were in the early stage of emphysematous cystitis, without symptoms and signs of sepsis and septic shock. As a result, they were effectively treated and presented a satisfactory prognosis. In the present case report, we highlighted the importance of targeted antibiotic therapies for emphysematous cystitis (15).

Overall, the diagnosis of EC can rely on abdominal CT to identify gas formation in and around the bladder wall. Through our case reports, we illustrated the typical characteristics and risk factors for EC, which should be highly suspected in older adults with diabetes mellitus and unknown causes of fever and abdominal pain. Once abdominal CT gas manifestations combined with positive urine microscopy and bacterial culture confirm the diagnosis of EC, early comprehensive intervention including anti-infection therapy, bladder irrigation and underlying disease control is of great significance to prevent disease aggravation, reduce mortality and improve patient prognosis. Combined treatment of surgical procedures is optional for EC, if necessary.

## Data Availability

The original contributions presented in this study are included in this article/supplementary material, further inquiries can be directed to the corresponding author.
